# The Functional Neuroanatomy of Developmental Dyslexia Across Languages and Writing Systems

**DOI:** 10.3389/fpsyg.2020.00155

**Published:** 2020-02-05

**Authors:** Fabio Richlan

**Affiliations:** Centre for Cognitive Neuroscience, Department of Psychology, Paris Lodron University of Salzburg, Salzburg, Austria

**Keywords:** brain, development, dyslexia, language, magnetic resonance imaging, orthography, reading, writing

## Abstract

The present article reviews the literature on the functional neuroanatomy of developmental dyslexia across languages and writing systems. This includes comparisons of alphabetic languages differing in orthographic depth as well as comparisons across alphabetic, syllabic, and logographic writing systems. It provides a synthesis of the evidence for both universal and language-specific effects on dyslexic functional brain activation abnormalities during reading and reading-related tasks. Specifically, universal reading-related underactivation of dyslexic readers relative to typical readers is identified in core regions of the left hemisphere reading network including the occipito-temporal, temporo-parietal, and inferior frontal cortex. Orthography-specific dyslexic brain abnormalities are mainly related to the degree and spatial extent of under- and overactivation clusters. In addition, dyslexic structural gray matter abnormalities across languages and writing systems are analyzed. The neuroimaging findings are linked to the universal and orthography-dependent behavioral manifestations of developmental dyslexia. Finally, the present article provides insights into potential compensatory mechanisms that may support remediation across languages and writing systems.

## Introduction

Developmental dyslexia is a disorder characterized by severe and persistent problems in the acquisition of literacy. Performance in reading skills is markedly below the age-adequate norm – in the absence of problems regarding intelligence, motivation, vision, or educational environment (American Psychiatric Association, 2013; World Health Organization, 2016). It has become evident from numerous studies that developmental dyslexia may not be viewed as a simple, single-trait disorder, that is, no single behavioral phenotype can be considered as a “typical” manifestation of dyslexia. There are problems in diverse aspects of literacy including reading fluency, accuracy, comprehension, and/or spelling, and people affected by dyslexia often present a mixture of different severities of these problems (e.g., Lyon et al., 2003). In addition, problems in learning to read are often comorbid with atypical or delayed oral language development (e.g., Catts et al., 2008; Peterson et al., 2009), writing disabilities, attention-deficit hyperactivity disorder (ADHD), and math disabilities/dyscalculia (e.g., Landerl and Moll, 2010; Willcutt et al., 2010).

Using neuroimaging techniques such as functional magnetic resonance imaging (fMRI), cognitive neuroscientific research has identified brain circuits crucially involved in typical and dyslexic reading. These studies have converged on a coarse functional neuroanatomical model of reading and developmental dyslexia. The model proposes abnormal brain activation in dyslexic readers in the left posterior temporo-parietal (TP) cortex (middle temporal gyrus, superior temporal gyrus, supramarginal gyrus, and angular gyrus), the left occipito-temporal (OT) cortex (inferior temporal gyrus and fusiform gyrus), and the left frontal cortex (inferior frontal gyrus and precentral gyrus).

As identified by meta-analyses, the most consistent finding is dyslexic underactivation relative to typical readers in the left TP and OT cortex. In addition, dyslexic underactivation was identified in the left inferior frontal gyrus (IFG) and dyslexic overactivation in the adjacent left precentral gyrus (Richlan et al., 2009, 2011; Martin et al., 2016; Hancock et al., 2017). Occasional reports on other bilateral cortical, subcortical, and cerebellar dyslexic deficits are not supported by the meta-analyses. Obviously, these dyslexic activation abnormalities depend largely on the utilized functional activation tasks during brain scanning, which are often targeted at providing evidence in favor of a specific neurocognitive deficit theory of dyslexia.

Although we have convincing evidence that the functioning of the above mentioned left TP, OT, and IFG cortical regions is altered in developmental dyslexia during reading and reading-related tasks, it is still an open question how the presumed functional and gray matter (GM) structural impairments in these regions lead to the severe and persistent reading problems of dyslexic readers. In other words, the question is not so much of whether and if so, where in the brain dyslexic abnormalities exist, but rather on how these brain regions might underlie reading- and spelling-related cognitive processes in typical and dyslexic readers. The present review article aims at providing an integrative overview and synopsis of the functional and structural brain abnormalities in dyslexic readers across languages and writing systems.

Specifically, the goal here is to focus on functional activation and GM structure; and on the commonalities and differences in these measures in developmental dyslexia across languages and writing systems. First, the functional neuroanatomy of developmental dyslexia across alphabetic languages differing in orthographic depth will be discussed. Second, the neurobiology of developmental dyslexia will be compared across alphabetic, syllabic, and logographic writing systems. Third, GM structural brain abnormalities in developmental dyslexia will be discussed. Finally, there will be a section on potential compensatory mechanisms that may support remediation across languages and writing systems.

Research on the relationship between functional activation and GM structure and their effects on reading development is of crucial importance but still scarce. Therefore, innovative approaches using intervention studies and longitudinal research will also be discussed. With respect to functional and structural connectivity in developmental dyslexia – which is beyond the scope of the present review – the reader is referred to other recent studies and meta-analyses (e.g., Ben-Shachar et al., 2007; Cao et al., 2008, 2017; van der Mark et al., 2011; Vandermosten et al., 2012; Koyama et al., 2013; Dehaene et al., 2015; Olulade et al., 2015; Schurz et al., 2015; Alvarez and Fiez, 2018).

## The Functional Neuroanatomy of Developmental Dyslexia Across Alphabetic Languages Differing in Orthographic Depth

Orthographic depth (OD) (i.e., the complexity, consistency, or transparency of grapheme-phoneme correspondences in written alphabetic language) (Frost et al., 1987) is a well-known factor influencing the acquisition of fast and accurate reading (Seymour et al., 2003; Landerl et al., 2013). Correspondingly, the behavioral manifestations of developmental dyslexia vary as a function of OD. Specifically, inaccurate mapping from graphemes to the corresponding phonemes is a particular hallmark of developmental dyslexia in irregular or deep orthographies – especially for English. On the contrary, persistent slow and dysfluent word recognition is a universal characteristic of developmental dyslexia across all alphabetic orthographies. Here we examine the question of how the different behavioral manifestations of developmental dyslexia are reflected in the functional neuroanatomical patterns identified by brain imaging studies.

The predominant view proposed a “cultural diversity and biological unity” account of developmental dyslexia, claiming a universal neurocognitive basis of the disorder across languages. This position was based on a seminal PET study comparing the brain activation of Italian, French, and English adult dyslexic readers in response to explicit and implicit reading tasks (Paulesu et al., 2001). The universal neurobiological substrate of developmental dyslexia across languages was reflected in underactivation (relative to typical readers) in a large left hemisphere cluster comprising the superior temporal gyrus (STG), middle temporal gyrus (MTG), inferior temporal gyrus (ITG), and middle occipital gyrus (MOG). Crucially, no orthography-specific effects in reading-related brain activation were identified in the direct statistical comparison of the dyslexic readers from the three languages varying in OD.

A qualitative summary and critical discussion of the Paulesu et al. (2001) study and more recent cross-linguistic brain imaging studies provided additional orthography-specific predictions regarding the degree and spatial extent of dyslexic under- and overactivation clusters relative to typical readers (Richlan, 2014). Together with the universal dysfunctions in core regions of the left hemisphere reading network (Pugh et al., 2005; Richlan, 2012; Martin et al., 2015), the presumed orthography-specific effects were derived from different functional neuroanatomical models of developmental dyslexia and dependent on the particular characteristics and processing demands of the language. In addition to differences in regional brain activation, deep orthographies (DO) and shallow orthographies (SO) were proposed to be associated with differences in the functional and effective connectivity between brain regions (Schurz et al., 2015).

Consequently, Martin et al. (2016) used coordinate-based meta-analysis in order to investigate the universal and orthography-specific predictions regarding dyslexic brain activation. Specifically, commonalities and differences of dyslexic functional brain abnormalities between alphabetic languages varying in OD were objectively quantified by comparing foci of under- and overactivation in dyslexic readers relative to typical readers as reported in 14 studies in DO (English) and in 14 studies in SO (Dutch, German, Italian, Swedish). The in-scanner activation tasks used in these 28 studies included silent reading, reading aloud, (phonological) lexical decision, rhyme judgment, semantic judgment, and sentence comprehension. Importantly, the two sets of studies in DO and SO, respectively, were balanced regarding the number of tasks that explicitly required phonological processing. For an in-depth discussion on the effects of task nature and task difficulty – which are difficult to control for in coordinate-based meta-analyses – we refer to the original publication (Martin et al., 2016).

As predicted from the cross-language literature (Paulesu et al., 2001), universal reading-related dyslexic underactivation was identified in the left OT cortex including the fusiform gyrus (FFG), inferior occipital gyrus (IOG), ITG, and MTG. Specifically, eight of 14 and nine of 14 studies contributed one or more activation foci in this region for DO and SO, respectively. The large left posterior cluster of overlapping underactivation in both DO and SO relative to typical readers also included the posterior-to-anterior gradient of the visual word form system (Dehaene and Cohen, 2011; Taylor et al., 2019). These regions can be regarded as the most consistently reported regions of dyslexic underactivation relative to typical readers in alphabetic orthographies – irrespective of OD, in-scanner activation task, and age of participants (the mean age of the participants in the 28 included studies ranged from 8 to 30 years).

The direct statistical comparison between the two sets of fMRI studies revealed higher convergence of dyslexic underactivation relative to typical readers for DO compared with SO in the bilateral inferior parietal cortex. Interestingly, this abnormality was no longer found when foci reported with stronger dyslexic task-negative activation (i.e., task-related deactivation relative to the resting baseline) were not included in the meta-analysis. Furthermore, higher convergence of dyslexic underactivation relative to typical readers for DO compared with SO was found in the triangular part of the left inferior frontal gyrus (IFG), the left precuneus, and the right STG. Higher convergence of dyslexic overactivation relative to typical readers was identified in the left anterior insula.

Higher convergence of dyslexic underactivation for SO compared with DO was identified in the left FFG, left TP cortex, the orbital part of the left IFG, and left frontal operculum. On the contrary, higher convergence of dyslexic overactivation relative to typical readers was found in the left precentral gyrus. In sum, the findings are in line with the view of a biological unity of developmental dyslexia – with a core deficit in the left OT cortex and additional orthography-specific variations. Different patterns of reading-related dyslexic overactivation are assumed to reflect different compensatory mechanisms across languages. The results of the meta-analysis by Martin et al. (2016) are summarized in Table 1.

**TABLE 1:** Brain abnormalities in developmental dyslexia identified in representative studies.

**Region**	**Functional abnormalities in alphabetic orthographies (Martin et al., 2016)**	**Functional abnormalities in syllabic/logographic writing systems**	**Structural abnormalities**
L occipitotemporal cortex	Underactivation in both deep and shallow orthographies	Underactivation in Chinese (Siok et al., 2004; Hu et al., 2010)	
B inferior parietal lobule	Higher underactivation in deep (due to stronger deactivation)		
L inferior frontal gyrus, triangular	Higher underactivation in deep	Overactivation in Chinese (Siok et al., 2004)	
L precuneus	Higher underactivation in deep		
R superior temporal gyrus	Higher underactivation in deep		Reduced gray matter volume in alphabetic (Richlan et al., 2013)
L anterior insula	Higher overactivation in deep		
L fusiform gyrus	Higher underactivation in shallow	Underactivation in Chinese (Siok et al., 2004; Hu et al., 2010)	
L temporoparietal cortex	Higher underactivation in shallow	Underactivation in Chinese (Hu et al., 2010)	Reduced gray matter volume in alphabetic (Richlan et al., 2013; Eckert et al., 2016)
L inferior frontal gyrus, orbital	Higher underactivation in shallow		Reduced gray matter volume in alphabetic (Eckert et al., 2016)
L frontal operculum	Higher underactivation in shallow	Underactivation in Chinese (Siok et al., 2004)	
L precentral gyrus	Higher overactivation in shallow		
L middle frontal gyrus		Underactivation in Chinese (Siok et al., 2004; Hu et al., 2010)	Reduced gray matter volume in Chinese (Siok et al., 2008)
L dorsal inferior frontal gyrus		Underactivation in Chinese (Cao et al., 2017)	
R cerebellum			Reduced gray matter volume in alphabetic (Eckert et al., 2016)

Importantly, common dyslexic underactivation in alphabetic orthographies was found in the left OT cortex, including the visual word form system. The universal left OT cortex dysfunction, most probably reflecting the phonological speed deficit characteristic of developmental dyslexia, is in line with evidence showing that in typical readers this area subserves both lexical whole-word recognition and sublexical serial decoding (e.g., Richlan et al., 2010; Schurz et al., 2010; Wimmer et al., 2010; Schuster et al., 2016) – at least in the studied alphabetic orthographies.

## The Neurobiology of Developmental Dyslexia in Alphabetic, Syllabic, and Logographic Writing Systems

In addition to the functional neuroimaging studies on reading and dyslexia in alphabetic orthographies, there have been studies on reading in syllabic (e.g., Japanese Kana), morpho-syllabic (e.g., Japanese Kanji), and logographic (e.g., Chinese) writing systems. In their meta-analysis of these studies on typical readers, Bolger et al. (2005) identified convergent reading-related activation in all of the above writing systems in a core network of the left STG, IFG, and OT cortex. A similar network of brain regions was found to show common activation across reading in Spanish, English, Hebrew, and Chinese (Rueckl et al., 2015). Accordingly, the brain activation abnormalities exhibited by dyslexic readers can probably be expected in similar regions across all writing systems. Direct evidence for this expectation, however, is still scarce.

The separate reading-related activation patterns of the different writing systems also varied to a certain extent, particularly regarding the spatial configuration of the activation clusters. Specifically, the meta-analysis by Bolger et al. (2005) identified divergence in the left STG (with more consistent activation for alphabetic and syllabic writing systems), and in the left IFG and right OT cortex (with more consistent activation for Chinese). The stronger activation for the alphabetic and syllabic writing systems in the left STG was ascribed to the fact that the written symbols are mapped to more fine-grained speech sounds (phonemes and syllables), as opposed to whole-word phonology in Japanese Kanji and Chinese. The stronger activation for Chinese in the left IFG was associated with higher demands on integrated processing of semantic and phonological information, which is required for unambiguous word recognition due to the high number of homophones in Chinese.

The first evidence for a specific brain dysfunction in Chinese dyslexic reading that was previously not reported for alphabetic writing systems was put forward by Siok et al. (2004). Their fMRI study found significant dyslexic underactivation in the left middle frontal gyrus (MFG) in Chinese children during both homophone judgment and lexical decision tasks. Accordingly, it was argued by the authors that fluent Chinese reading relies on the integrity of the left MFG as a main hub for the coordination and integration of information in verbal and spatial working memory and that developmental dyslexia results from a failure of this brain region (Perfetti et al., 2006).

The left MFG was also identified in a direct cross-linguistic comparison between dyslexic and typical readers of Chinese and English using a semantic word matching task (Hu et al., 2010). Despite brain activation differences between Chinese and English typical readers, the dyslexic readers of both writing systems showed a similar pattern of underactivation compared with the typical readers in the left MFG, left TP cortex, and left OT cortex. That is, in contrast to previous studies (see Table 1), even the English dyslexic readers were identified as exhibiting underactivation in the left MFG. Therefore, the functional neuroanatomical signature of developmental dyslexia in Chinese and English seems to be more similar than originally proposed by Siok et al. (2004) and reflected in underactivation of a common network including left (middle) frontal, TP, and OT regions – at least when a semantic processing task is used during brain scanning.

A remarkably similar brain network was identified by Cao et al. (2017) using an auditory rhyme judgment task. Specifically, they found that Chinese children with developmental dyslexia exhibited underactivation of a left dorsal IFG region relative to both age-matched and reading performance-matched control participants. Although anatomically labeled as left IFG, the maximum of the activation cluster was in close proximity to the left MFG with an Euclidean distance of only 16 mm and 8 mm to the peaks reported by Hu et al. (2010) and Siok et al. (2004), respectively. This left IFG dysfunction was associated with a phonological processing deficit of dyslexic readers that correlated with the severity of reading problems. Furthermore, analyses of functional connectivity identified weaker connections between the left IFG and left FFG and between the left STG and left FFG in dyslexic readers compared with the control participants. These findings were interpreted as reflecting a problem in the connection of orthography and phonology in Chinese developmental dyslexia.

## Structural Brain Abnormalities in Developmental Dyslexia Across Languages

Seminal neurological examinations on the neural basis of acquired reading problems were already conducted in the nineteenth century (Dejerine, 1891, 1892). In the case of developmental reading problems, neurological studies in the 1970s and 1980s were based on histological brain examinations. For example, Galaburda and Kemper (1979) identified reduced left-right asymmetry of the planum temporale in a post-mortem brain examination of a dyslexic reader. Further studies by Galaburda et al. (1985) and Humphreys et al. (1990) reported additional structural abnormalities such as neuronal ectopias and architectural dysplasias in the left TP cortex of four more dyslexia cases.

The advent of modern-day neuroimaging technology and the development of Voxel-Based Morphometry (VBM; Ashburner and Friston, 2000), enabled the automatic and objective analysis of brain structure *in vivo*. In short, VBM provides a measure of local GM volume or density of a voxel. It is an established method in cognitive neuroscience and has been used to investigate pre-reading children with a familial risk for dyslexia (e.g., Raschle et al., 2011, 2015; Black et al., 2012), dyslexic children (e.g., Eckert et al., 2005; Hoeft et al., 2007; Kronbichler et al., 2008; Krafnick et al., 2014; Jednoróg et al., 2015), and dyslexic adults (e.g., Brown et al., 2001; Brambati et al., 2004; Silani et al., 2005; Steinbrink et al., 2008; Pernet et al., 2009).

Regarding structural abnormalities in the brain of Chinese dyslexic readers, first evidence was again reported by Siok et al. (2008). Similar to the region identified with dyslexic underactivation in their previous functional MRI study (Siok et al., 2004), they found reduced GM volume in the left MFG of dyslexic children. Crucially, no other cortical or subcortical regions exhibited differences in GM volume between dyslexic and typical readers of Chinese, even in a sensitive regions-of-interest analysis focused on the left MTG, TP, and OT cortex.

A recent study (Qi et al., 2016) examined large-scale brain networks in Chinese dyslexic children. In their analysis of structural T1-weighted MRI data they distinguished between two complementary measurements of neuroanatomy in order to disentangle early congenital effects from later developed effects. Specifically, whereas the measurement of cortical surface area is thought to be sensitive to prenatal development, the measurement of cortical thickness is thought to be more sensitive to postnatal development. The Chinese dyslexic children exhibited abnormalities in both measurements, in the sense that the structural brain networks of the dyslexic children were more bilateral (i.e., less lateralized), more distributed in anterior brain regions, and less distributed in posterior brain regions compared with the typically reading children.

Due to the substantial number of existing VBM studies on dyslexia, objective coordinate-based meta-analyses were used in order to identify and specify stable effects across studies (e.g., Richlan et al., 2013). As shown in Table 1, consistent GM volume reduction in developmental dyslexia in alphabetic orthographies was identified in the right STG and in the left superior temporal sulcus (STS). The robustness of these findings, however, was limited as convergence across studies was relatively weak with only about half of the studies contributing to the meta-analytic clusters.

The limited convergence across studies was recently critically examined in more detail by Ramus et al. (2018). They argued that most VBM studies on developmental dyslexia are based on relatively few and relatively heterogeneous participants, leading to a high number of false positive rates in the primary literature and, therefore, little replicability of results across independent studies. This issue concerns cross-linguistic comparisons probably even more, with additional sources of heterogeneity including different assessment tools, educational systems, and socio-demographic factors.

Nevertheless, the findings of our meta-analysis found plausible support in other structural neuroanatomical studies on reading and dyslexia. The right STG region was a focal point in a remarkable and unique study by Carreiras et al. (2009). In this study, the researchers investigated (ex-) illiterates who did (or did not) learn to read as adults. The main finding was that learning to read was accompanied by an increase in GM volume in bilateral TP and dorsal occipital regions. Concerning the meta-analysis on structural brain abnormalities in developmental dyslexia, this result indicates that the right STG GM volume reduction exhibited by dyslexic readers might reflect their reduced reading experience. Therefore, the GM volume reduction is a consequence rather than a cause of reading problems in developmental dyslexia.

Two VBM studies with pre-reading children, however, support a different interpretation of the right STG GM volume reduction. Specifically, Raschle et al. (2011), reported that children with a high family-risk for developmental dyslexia were identified as having reduced GM volume in both left and right TP cortex even before formal reading instruction. Likewise, Black et al. (2012) found that a family history of reading disability was related to a reduction in GM volume in the bilateral TP cortex of five to 6-year old beginner readers. In this age group, the structural brain abnormalities can hardly be explained by a reduced amount of reading experience.

While the GM volume reduction in the right STG was an unexpected finding of our meta-analysis, the GM volume reduction in the left STS was not. The left STS GM volume reduction is in line with a large body of evidence for left perisylvian cortical anomalies in dyslexia, as identified in the already mentioned post-mortem brain examinations (e.g., Galaburda et al., 1985) and in early brain imaging studies (Eliez et al., 2000). Crucially, a similar left temporal region was identified as showing GM volume reduction across Italian, French, and English adult dyslexic readers (Silani et al., 2005). More recently, the left STS was shown to be one of the most reliable regions identified with reduced GM volume in developmental dyslexia in a combined meta-analysis and multi-center study across different laboratories from the United States (Eckert et al., 2016).

In order to interpret the functional effect of left STS abnormality in developmental dyslexia, it is important to investigate its role in typical and disrupted language processing. Classically, neurological lesions of the left STS were linked to problems in speech comprehension (Wernicke’s aphasia). In more up-to-date conceptions on the neurology of speech and language (e.g., Hickok and Poeppel, 2007), the function of the left STS is associated with the representation and processing of multimodal phonological information. Therefore, it is recruited by both perceptual and productive speech processes, as well as by working memory processes involving phonological information. These cognitive functions are particularly crucial for a successful start at the beginning of literacy acquisition across languages.

Across different alphabetic orthographies, the left STS is assumed to play an important role in the integration of auditory and visual information (e.g., van Atteveldt et al., 2004; Blomert, 2011; Holloway et al., 2013; Richlan, 2019). Therefore, during skilled reading and especially during typical reading acquisition, it is recruited by self-reliant learning processes based on serial grapheme-to-phoneme conversion. The structural GM volume reduction in the left STS in developmental dyslexia might be related to problems in this sublexical self-teaching reading strategy. Specifically, it was proposed that dyslexic readers suffer from a disruption in the development of a brain system for efficient interactive processing of auditory and visual linguistic inputs (Blau et al., 2010). Taken together, the existing evidence suggests that left STS and right STG GM volume reductions are reliable neuroanatomical signatures of adult dyslexia across different alphabetic orthographies, which might exist even before the onset of formal reading instruction.

## Limitations and Future Directions

Cross-linguistic comparisons have proven to provide extremely valuable information on the neurobiology of reading and developmental dyslexia. The focus, up to now, was largely on the comparison of dysfunctions in the form of reading-related dyslexic underactivation relative to typical readers. In contrast, the patterns of dyslexic overactivation relative to typical readers were rarely compared across languages and writing systems. This is probably because there is larger inter-individual variability with respect to overactivation compared with underactivation in developmental dyslexia and, in turn, less consistency across studies (and activation tasks). From the results reported by Martin et al. (2016), it seems that OD plays a role in the consistency of dyslexic overactivation patterns, with English dyslexic readers exhibiting more heterogeneous patterns compared with dyslexic readers from SO. This leads to only a single meta-analytic cluster identified with overactivation in English dyslexic readers compared with seven meta-analytic clusters in dyslexic readers from SO.

In principle, the dyslexic overactivation patterns might be informative on potential compensatory mechanisms supporting language-specific or language-universal remediation strategies. First evidence (Martin et al., 2016; Cao et al., 2017; Hancock et al., 2017) points to an important role of the precentral gyrus possibly subserving such neural compensation. At least in alphabetic orthographies, this compensatory role was attributed to increased reliance on articulatory processing in dyslexic readers (Hancock et al., 2017), particularly for dyslexic readers from SO. Future studies across different languages and writing systems, however, are urgently needed to shed more light on this issue.

One way of providing this kind of evidence is via intervention studies and longitudinal research. These longitudinal brain imaging studies would also be helpful for a better understanding of the relationship between brain function and brain structure and their respective effects on reading development across languages. Unfortunately, such cross-linguistic longitudinal studies are extremely challenging to conduct and to analyze, and therefore, do not exist yet. Certainly, more fundamental research on the interplay between the developmental changes in brain function, brain structure and literacy acquisition is required in order to put forward comprehensive brain-based models of typical and dyslexic reading development.

## Conclusion

Across alphabetic writing systems, OD has an influence on the relative importance of different underlying cognitive processes required for fluent reading, and accordingly on the degree and spatial extent of brain activation clusters of typical readers. Consequently, the neuroanatomical dysfunctions of dyslexic readers are associated with an emphasis on different elements of the core reading network, reflected in stronger or weaker under- and overactivation relative to typical readers depending on OD. For example, in the case of the logographic Chinese writing system, a crucial role is assigned to the left MFG, which possibly subserves the working memory processes required for the successful recognition of written characters.

The existing evidence, up to now, suggests that the functional neuroanatomy of developmental dyslexia is similar across languages and writing systems, with some orthography-specific peculiarities. Specifically, underactivation (in dyslexic readers relative to typical readers) in core regions of the left hemisphere reading network including OT, TP, and IFG regions in response to reading or reading-related tasks seems to be a universal signature of developmental dyslexia. At least parts of the core network were also identified with structural neuroanatomical abnormalities in dyslexic readers – sometimes even before the onset of formal reading instruction (in children with a familial risk for developmental dyslexia). Consequently, these core regions are language-universal prime candidates to be targeted by intervention programs.

## Author Contributions

FR conceived and wrote the manuscript.

## Conflict of Interest

The author declares that the research was conducted in the absence of any commercial or financial relationships that could be construed as a potential conflict of interest.
